# Comment on Ulas et al. Prognostic Insights into Orbital Metastases: A Comprehensive Analysis of Clinical Features and Survival Outcomes. *Diagnostics* 2025, *15*, 2542

**DOI:** 10.3390/diagnostics16101534

**Published:** 2026-05-19

**Authors:** Zahra Naderi Beni, Afsaneh Naderi Beni, Ali Salehi

**Affiliations:** 1 Shahrekord University of Medical Sciences, Shahrekord 8816758915, Iran; 2 Isfahan Eye Research Center, Isfahan University of Medical Sciences, Isfahan 817467346, Iran

We read with great interest the comprehensive 20-year review of orbital metastases by Ulaş and co-authors [1]. The authors are to be commended for presenting valuable regional epidemiological and survival data on this rare condition.

We would like to offer the following constructive observations that may further contextualize their findings:

1. Given the relatively small cohort (n = 83) and particularly small subgroups for certain primary tumors, multivariate survival analyses or detailed histopathological/molecular subtyping (e.g., lobular versus ductal breast carcinoma or receptor status) would have been statistically inappropriate and prone to overfitting or spurious results. The authors appropriately exercised caution in this regard [2,3].
2. The absence of carcinoma of unknown primary (0%) in this tertiary referral series, while differing from some larger published cohorts (19–25%) [4,5], is consistent with several reports from specialized orbital centers where extensive systemic evaluation is routine (CUP rates 0–6%) [6,7,8]. This likely reflects referral patterns and diagnostic intensity rather than significant selection bias.
3. As the authors transparently acknowledged, heterogeneity of systemic and local treatments, small subgroup sizes, and limited statistical power preclude definitive prognostic conclusions [9]. We fully agree with their cautious interpretation.

We believe this work adds meaningful regional insights to the literature on orbital metastases.
